# The Institutional Library

**Published:** 1916-07-01

**Authors:** 


### THE INSTITUTIONAL LIBRARY.

**Pye's Surgical Handicraft.** Edited and largely re-written by W. H. Clayton-Greene, F.R.C.S. Seventh Edition. 1916. (Bristol: John Wright and Sons. Pp. 614. Price 15s. net.)

There is a vast deal of common surgical procedure that is not dealt with, or not fully dealt with, in full-dress text-books of surgery, and is seldom inquired after by examiners for surgical qualifications. This surgical handicraft, as Mr. Pye called it more than thirty years ago, the young surgeon has to pick up for himself on taking up a resident surgical appointment, and until he has learnt it he cannot do full justice to his patients. Some men naturally pick it up very quickly from their visiting staff's hints, from their ward and casualty-room sisters, and from their experience; others take longer in the process. One and all of them stand to gain immensely by a careful study of minor surgery; and we know of no book in which its principles and practice are more fully and more clearly enunciated as in this manual. Yet there are sections, even in this valuable work, in which improvement could be wished for. Twenty-nine lines upon bullet wounds, for instance, is scant allowance for war time, especially as the lessons of the war do not seem to have been considered. The description of spinal anæsthesia is very poor. The use of carbon dioxide snow for nævi is advocated, but no details whatever are afforded of how it should be procured or applied. The sections on syphilis are mutually contradictory, and want re-writing. The general impression is that not quite enough revision has been carried out in this edition.

**Diseases of the Nose and Throat, comprising Affections of the Trachea and Œsophagus.** By Sir St. Clair Thomson. Second Edition. 8vo. Pp. xvi.+858. With 22 Plates and 377 Figures. (London: Cassell and Co. 1916. Price 25s. net.)

Since its publication in 1912 St. Clair Thomson's "Diseases of the Nose and Throat" has taken a foremost place as an authoritative and exhaustive text-book of the subject, and we now welcome the appearance of a second edition. The progress of laryngology and rhinology has been rapid in these four years, and a work which does not include an account of the recent advances in the science is to some extent out of date. The new edition contains a short description of suspension-laryngoscopy, and an adequate account of intranasal dacryocystotomy, and of the intranasal route to pituitary tumours, though the latter gives the ordinary anterior incision for a submucous resection, and does not mention the various modifications designed to give more room. Courtenay Yorke's method of "nerve-blocking," to obtain anæsthesia of the superior laryngeal nerve, is fully considered; a watery solution of novocain is employed, and the author apparently finds this more efficacious in the dysphagia of tuberculous laryngitis than alcohol injection into the nerve on the thyro-hyoid membrane, for this older method is not described. Diathermy, though mentioned frequently, is nowhere given a description. The Killian operation for frontal sinusitis now takes a second place, the intranasal operation through the agger cells being first discussed: the technique and the instruments required are not, however, described with sufficient detail to be of great service to the rhinologist who wishes to attempt this operation. The index is a full one, the illustrations are numerous and excellent, and frequent references to authorities are given. The work throughout shows evidence, not only of care and accuracy, but of experience and authority, and will be useful alike to the student, the practitioner, and the specialist.

**Occupations.** By Sir Thomas Oliver, M.D., F.R.C.P. (Cambridge: The University Press. 1916. Pp. 110. Price 6s. net.)

This addition to the Cambridge Public Health Series is an elementary introduction to the study of the effects of occupation upon health and life. It is not too technical for general readers, and is hardly encyclopædic enough to be regarded as a medical text-book. As might be expected, the information provided by this well-known and expert teacher is well arranged and clearly expressed, though the English style of it is not faultless. From certain statistics on p. 64 it is made to appear that between the ages of twenty-five and sixty-five the mortality rate is two and one-third times as great amongst unoccupied males as amongst the occupied; inference, the dangers of idleness. But surely the real explanation of the figures is that in many cases it is the illness preceding death that accounts for the lack of occupation, not the latter that accounts for the illness?

**Pharmacy and Dispensing.** By C. J. S. Thompson. New Edition, thoroughly revised. (London: The Scientific Press, Ltd. Price 1s. net.)

To-day is the day of the woman dispenser. Ever since the commencement of the war, when young pharmacists began to leave the dispensary for active service, there has been a constant demand for trained women to take their places, and now that the Army Council has determined that in future preference shall be given to women dispensers in military hospitals, more and more certified women will be needed. This book is intended as a guide for nurses and other women desirous of qualifying for this occupation, and in preparing a new edition the author has been well advised to include a short chapter indicating the courses of study necessary for those who desire either to become fully qualified pharmacists or to obtain the Assistant's Certificate of the Society of Apothecaries. A more useful little book for those who are learning the art of dispensing it would be difficult to find. It must, naturally, be studied in conjunction with a course of practical instruction, and the knowledge so acquired by the learner must be supplemented by actual experience under proper supervision; for, just as no one can learn how to dispense without knowing something about the properties of the things dispensed—which knowledge can best be acquired from books—so no dispenser can ever become competent by the aid of books alone. There is no need to traverse the familiar chapters of a book that has passed through many editions, and it will suffice to say that they deal with all the difficulties a beginner is likely to encounter, and explain in a way that will be easily understood all the points a beginner ought to know.

**Children's Nursing.** By C. Seymour Yapp, Matron of the Poor-Law Hospital, Ashton-under-Lyne, Author of "Modern Medical and Surgical Nursing for Probationers." (London: Poor-Law Publications, Ltd., Furnival Street, E.C. Price 2s. 6d.)

This book is intended to supplement the author's previous work on general nursing, and she is able, therefore, to dispense with some of the more elementary facts which have already been dealt with in that volume. The result is a book which, though it is intended for probationers, will be read with great pleasure by all nurses, however experienced they may be. From the opening chapters, which deal with the care of healthy children, right on through the list of children's diseases and ailments, the author holds the reader's attention, and the impression left on the mind of at least one experienced nurse is that the writer is dealing with a subject that she has thoroughly mastered. Miss Yapp thinks that in a children's ward there should be a nurse for four children, and certainly that number will be needed if the children are to have all the care and mothering that she desires for them. The book is full of useful hints; for instance, that a child's toys should be tied to the cot to prevent them falling on the floor. In writing of infants' mothers Miss Yapp says, "You are all familiar with the type of patient so frequently brought in—the emaciated little skeleton, etc. I have sometimes found a tendency on the part of young nurses to blame the mother for the condition of these children, and whilst you are in training let me impress upon you that these mothers need your pity as much as the babes in the majority of cases. . . . The harassed mother who brings up her baby knows by the very expression of your face that you blame her, and I do assure you that her weary 'I have done my best' is often true. Do let me beg of you to extend your pity to the mother who has failed; make her *feel* that you think she has put up a good fight. This attitude is so necessary in a nurse, and will be your greatest asset when you go to work amongst them in their homes."

We entirely agree with Miss Yapp that "babies in a children's ward should be lifted from their cots in the children's ward, and nursed before the fire, if possible, at least four or five times a day for at least ten minutes." We should like to see this book in the hands of every probationer in both voluntary and Poor-Law hospitals.

**The Medical Annual, 1916.** Thirty-fourth Year. (Bristol: J. Wright and Sons. London: Simpkin, Marshall, Hamilton, Kent and Co. Pp. 800. Price 10s.)

Undisturbed by the war, this invaluable publication pursues the normal tenor of its ever-expanding usefulness as a cyclopædia of the year's progress. Naturally enough the war bulks somewhat large in its pages, for so much research has been done on war surgery and medicine, so many new ideas have been ventilated in war hospitals, that any publication of this sort must necessarily devote a large share of attention to war matters. New splints for fractures, new antiseptics, new developments of wound surgery are fully dealt with; and the articles on typhus fever, cerebro-spinal fever, paratyphoid fever, soldier's heart, and other medical subjects show that the physicians have been almost as energetic as the surgeons. A special section is devoted to military medical matters; the clinical part of this is entrusted to Deputy-Surgeon-General A. G. Wildey, R.N., and the bacteriological part to Dr. A. C. Inman. The book is compiled throughout on the same lines and with the same thoroughness as have long been familiar to the medical profession; it is as full a half-sovereign's worth this year as it has ever been, and that is saying a good deal.

**Letters from Another Battlefield.** (London: Erskine Macdonald. Price 1s. net.)

These letters from a sanatorium belong to the subjective, Arthur Benson school of penmanship. They record the interest of a bed-bound patient in the vista which opens through his or her window into the world of Nature outside; the importance which, in a life of routine, attaches to every incident of the day and makes every nurse or doctor or visitor eventful. We all suffer from what may be called the will to self-justification, but this preoccupation is especially felt by patients whose desires are limited, whose external interests are few, and who, more than the rest of us, have to dig what they can out of themselves. In the cheerfulness of Stevenson, another consumptive, there is something feverish. There is a hectic spot in his mirth. His enamelled style itself betrays the invalid. There is a similar note here, but a touch of sentiment is given to the letters by the love story, with its unhappy ending, which is suggested rather than told as the correspondence proceeds. Judging by the success which attends Mr. Benson's innumerable variations on the same theme, this collection may easily be popular. It is cultured, pensive, sentimental, with the familiar background of sickness, striving against the intangible, and so on; in short, an example, we suppose, of *belles-lettres*.

### BRITISH NURSING.

**Macmillan and Another v. Nursing Press, Limited, and Others.** In the High Court of Justice, King's Bench Division, Royal Courts of Justice, London, Tuesday, March 14, 1916, before Mr. Justice Ridley and a Special Jury. (Published by the Proprietors of The Hospital, The Hospital Building, 28-29 Southampton Street, Strand, London, W.C. 1s. net.

An authentic history of the rise and development of trained nurses in Great Britain is yet to seek. The last forty years, and especially the last twenty years, have been full of developments, controversy, extensions, improvements, and progress. Indeed, the changes have been so many, and in one sense so kaleidoscopic, as to make it difficult for working nurses who are usually busy people to keep track of all that has gone on and is still proceeding. In such circumstances the trial of the action of which a full report is now available has a special interest, for it contains accurate information and facts which have heretofore been known only to the few, though they ought to be studied and grasped by every member of the nursing profession. We have heard much of the quack nursing Press and of the assertion, which constant reiteration has resulted in making current in otherwise well-informed nursing centres in the United States of America, to the effect that the men who have been associated with training schools or have taken a leading position in movements having for their object the development of the nursing profession in Great Britain, have had neither knowledge nor genuine interest in the nurse as such, but only a hungry greed to make what the American people call dollars for themselves at the expense of the nurses. These untruths and others, as shameful as they are false, have been continuously uttered and printed by a few interested people who have had axes to grind. We recommend every impartial person to obtain and read this full report of the trial of Macmillan and another *v.* Nursing Press, Limited, and others. It is difficult to credit the length to which human beings obsessed by fixed aims and ideas, however false, may be carried. A full report of a trial, with the cross-examination of witnesses, is sometimes a revelation well worth the time devoted to its perusal. This report may prove to be of enlightening value to the American Press and to the leaders of nursing in the United States, on the Continent of Europe, and in other countries where trained nurses are, in fact, to be found.

